# Synergistic effect of low-dose gamma radiation and/or clove oil loaded on silica nanoparticles on regeneration of human gingival fibroblast cell line: an in vitro study

**DOI:** 10.1007/s10266-025-01198-y

**Published:** 2025-09-18

**Authors:** Asmaa M. Abdeen, Mustafa Gharib, Ibrahim Y. Abdelrahman

**Affiliations:** 1 Department of Oral Biology, Faculty of Dentistry, Minia University, Minia, 61511 Egypt; 2 Department of Radiation Biology, National Centre for Radiation Research and Technology (NCRRT), Egyptian Atomic Energy Authority (EAEA), Nasr City, Cairo, 11765 Egypt

**Keywords:** Clove oil, Silica nanoparticle, Gingival fibroblasts, Low-dose gamma radiation, Wound healing

## Abstract

In light of the growing use of natural products in medicinal therapy, this study evaluated the proliferative effects of clove oil (CO) in two forms—native and loaded onto silica nanoparticles (CO@SNPs)—on Human Gingival Fibroblasts (HGFs), both singly and in combination with low-dose gamma irradiation (0.25 Gy). Silica nanoparticles were chemically synthesized and loaded with clove oil, with thorough characterization conducted using dynamic light scattering (DLS) for particle size, zeta-potential measurements, Fourier transform infrared spectroscopy (FTIR), and transmission electron microscopy (TEM). The EC50 values of CO and CO@SNPs were determined to be 1.88 µmol/mL and 0.172 µmol/mL, respectively, demonstrating a superior proliferative efficacy of the CO@SNPs nanoformulation. The biological effects were assessed using the MTT assay for cell viability, Trypan blue exclusion for cell count, qPCR for gene expression of c-Myc, MAPK, and NF-κB, and antioxidant capacity assays (MDA and catalase activity). Moreover, the in vitro wound-healing scratch assay showed enhanced fibroblast migration and proliferation, with relative wound density reaching 48% and 62% for CO@SNPs alone and combined with low-dose gamma irradiation, respectively. Further analysis through immunofluorescence staining of TGF-β and hematoxylin staining confirmed cell proliferation. Our results showed that loading CO on silica nanoparticles (CO@SNPs) were significantly more efficient than native clove oil in promoting cell proliferation and wound healing, with the combined treatment of CO@SNPs and low-dose gamma irradiation producing the most pronounced regenerative effect. These findings suggest that CO@SNPs, especially when combined with low-dose gamma irradiation, hold significant promise for enhancing oral tissue regeneration.

**Graphical abstract:**

- **Nanotechnology-enhanced clove oil delivery**: The present study successfully encapsulated clove oil (CO) in silica nanoparticles (CO@SNPs), which improved its stability and controlled release and provided its therapeutic efficacy.
- **Antioxidant and wound-healing properties**: CO@SNPs significantly reduced oxidative stress markers (e.g., malondialdehyde levels decreased by 93%) and enhanced antioxidant enzyme activity (catalase activity increased by 83%) in human gingival fibroblasts (HGFs) under low-dose radiation conditions.
- **Improved cellular proliferation**: CO@SNPs promoted HGF proliferation and wound closure in vitro, demonstrating a synergistic effect when combined with low-dose gamma irradiation, achieving optimal healing outcomes.
- **Biocompatibility and safety**: CO and CO@SNPs exhibited a high cell viability with low cytotoxicity at effective concentrations, supporting their safe application in clinical settings.
- **Implications for dentistry and beyond**: These findings highlight the potential of CO@SNPs in nanodentistry and other biomedical applications for managing oral and tissue wounds efficiently.

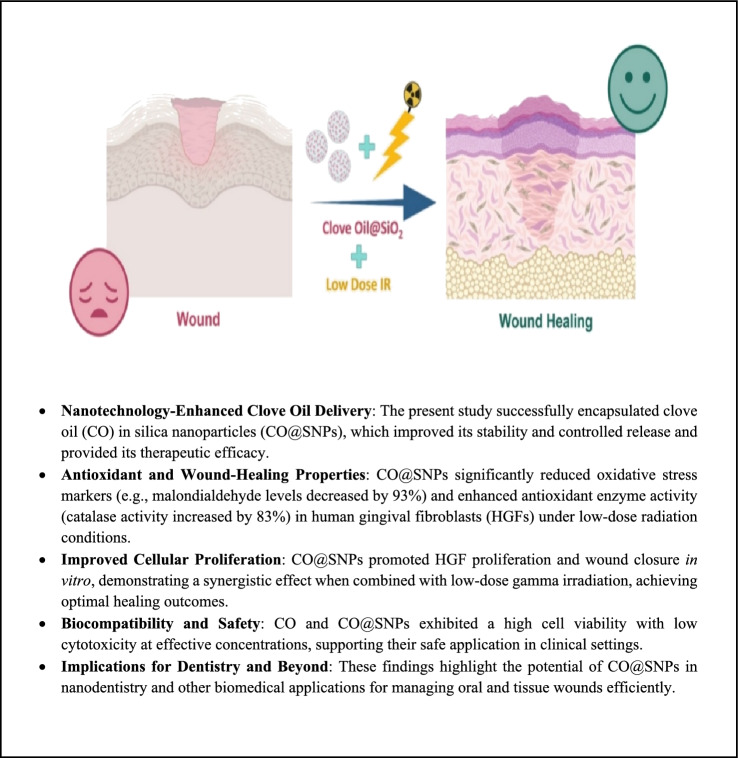

## Introduction

Over the past three decades, there has been a significant rise in the use of herbal medications and supplements, with at least 80% of people using them as complementary and alternative medicine globally [1].

Essential oils (EOs), which are aromatic compounds with complex combinations of terpenes, terpenoids, and phenylpropanoids, are naturally produced by plants as secondary metabolites. They are also regarded by the Food and Drug Administration (FDA) as generally recognized as safe (GRAS) [2].

Their composition varies and is a significant factor in determining their activity; phenolic groups make them more potent in terms of their antibacterial and antioxidant properties [3]. Oils containing phenolic groups include those from oregano, peppermint, cloves, basil, and cinnamon [4].

EOs have long been utilized for their biological effects, which include anesthetic, antibacterial, analgesic, sedative, anti-inflammatory, and spasmolytic qualities [5]. The cytotoxic and antiproliferative properties of a number of essential oils (EOs) have been studied in the past ten years, both in vitro on various cell lines and in vivo using experimental animals, in an effort to explore their potential utility as an alternative or supplemental anticancer treatment [5].

However, several explanations have been put out for the documented cytotoxic effects of EOs or their constituents, such as cell cycle arrest, loss of vital organ function, and induction of cell death by necrosis and/or apoptosis [6].

The low molecular weights and lipophilic characteristics of Eos’ component parts enable them to pass through cell membranes by changing their phospholipid layers, causing the membrane to become more fluid, and causing ion and cytoplasmic content to leak out. Cellular membrane disruptions can lead to a variety of negative effects, including decreased ATP generation, altered pH gradients, and loss of mitochondrial potential. Additionally, EOs influence the redox state of cells by acting as antioxidants [7].

The most significant essential oil that is derived from the spice *Syzygium aromaticum* (*S*. *aromaticum*) is clove oil (CO). Among other essential oils, clove oil has drawn a lot of attention due to its potent anti-aging, anti-inflammatory, antioxidant, and pathogenic organism-fighting qualities. The reason for these actions is that the clove flower buds contain more than 18% volatile oil, which contains unsaturated phenolic chemicals, primarily eugenol, iso-eugenol, vanillin, eugenyl acetate, and β-caryophyllene [8].

The dental community has also seen the impact of clove oil as a regular ingredient in treating dental problems. Clove oil remedies also have a long history of use for gingiva and tooth problems. To name a few, dating back to the third century b.c., Chinese emperors were known to utilize CO as a breath refresher. CO was mentioned in ancient Hindu writings for dental purposes. Hippocrates, known as the “Father of Medicine”, was taught by Avicenna, who used CO capsules to cure teeth decay and gingival disease. Since the 1800s, root canal therapy and other more advanced dental procedures have been made possible by the germ-killing compounds found in CO [9]. In particular, CO’s eugenol molecule inhibits the growth of almost all intraoral disease-causing bacteria while sparing helpful bacteria from damage. Eugenol is also analgesic and antiseptic. Throughout the years, its topical analgesic qualities have led to its application in tooth fillings and dental cement. In places where access to pharmaceutical topical anesthetics is restricted due to cost or availability, clove gel can offer dentists a better option than benzocaine for topical anesthesia in their routine practices, particularly when treating pediatric patients [10].

Exhilaratingly, nanotechnology applications have a powerful impact on medicinal applications where it is used as a carrier for some natural ingredients to help accelerate the receiving of their target and increase their efficiency [11, 12].

Gamma radiation has a double-edged sword, where a high dose of radiation has a harmful effect on living cells, but low doses of radiation have a hormesis effect on living cells, which means that the tiny doses of gamma radiation have beneficial effects on cells, including activation and adaptive response of repairing biochemical and molecular mechanisms, which do not take place in the absence of radiation [13, 14].

The new technology of bionanoapplications in the medicinal field has an important role in increasing the efficiency of drugs and natural active ingredients in their application. The nanosize of some nanoparticles increases the pharmacological functions regarding pharmacodynamics and pharmacokinetics specifications. Different nanoparticles work as carriers to active ingredients, causing a high effect on target cells; one of those nanoparticles is mesoporous silica nanoparticles [15].

Therefore, this study aims to assess the proliferative impact of both native clove oil (CO), and clove oil loaded in silica nanoparticles (CO@SNPs) on the growth of Human Gingival Fibroblasts (HGFs), whether they are non-irradiated or have been subjected to a low dose of ionizing radiation.

## Materials and methods

### Ethical statement

This study was approved by the Research Ethics Committee, National Centre for Radiation Research and Technology (NCRRT), Cairo, Egypt. The committee approval number was 36A/23.

- Clinical trial number: not applicable.

### Material

Clove oil was obtained from Ayurveda Co., Egypt. Tetraethyl orthosilicate, ADVENT Co., Tri-Ethanolamine, SDFCL Co., Cetyltrimethylammonium bromide 98% (CTAB), ADVENT Co., The present study was conducted at NCRRT, Cairo, Egypt.

### Methods

#### Preparation of clove oil-loaded mesoporous silica nanoparticles

**Synthesis of silica nanoparticles (SNPs)**

Silica nanoparticles were synthesized following a slightly modified version of a previously published protocol by [16]. Briefly, 20 mL of DI water was used to dissolve 2 g of CTAC and 0.04 g (0.04 mL) of TEA. The mixture was then vigorously stirred for 1 h at 95 °C in a silicone oil bath (prepare 100 mL). After an hour, 1.5 mL of TEOS was added to the dispersion dropwise at a rate of 1 mL/min (20 μL/s). For an additional hour, this mixture was vigorously agitated at 95 °C. After letting the mixture cool, the SNPs were separated into pellets using centrifugation (20,000×*g*, 15 min). Following many ethanol washes, the particles were baked for a whole night at 60 °C to dry them off. To totally eliminate the surfactant, the dry pellets were crushed and calcined for 7 h at 550 °C in a muffle furnace (Thermoscientific, Australia) [17].

**Clove oil loading on silica nanoparticles (CO@SNPs)**

100 mg of clove oil was dissolved in 2 mL of acetonitrile in a glass vial. Then, 100 mg of SNPs were added, and the caps of vials were closed. The mixtures were incubated on a rotary shaker at 200 rpm at room temperature for 48 h. After that, the resulting loaded nanoparticles were centrifuged, dried at 50 °C, and collected [18].

#### Characterization of clove oil-loaded silica nanoparticles (CO@SNPs)

The transmission electron microscopy (TEM, 1230, with an accelerating voltage of 100 kV, JEOL, Japan), dynamic light scattering (DLS, Zeta sizer Nano ZN, Malvern Panalytical Ltd, United Kingdom), and Fourier transform infrared (FTIR, Bruker Alpha FTIR Spectrometer, NAWAH Co.) techniques were employed to characterize the nanoparticles, including their size, morphology, charge, and surface functionality.

#### Cell culture

The Human Gingival Fibroblasts (HGFs) cell line was certified at ATCC, and their manipulation was placed at the Tissue Culture Unit of Global Lab, Cairo, Egypt. One day before conducting the experiment, the HGFs were seeded in two T-25 culture flasks. An average of 0.7 × 106 HGFs were seeded in 15 mL of Dulbecco’s Modified Eagle Medium (DMEM) (Gibco, Thermoscientific, Germany) containing 10% fetal bovine serum (FBS) (Gibco, Thermoscientific, Germany) and 1% of penicillin G sodium (10,000 UI), streptomycin (10 mg), and amphotericin B (25 μg) (PSA) (Gibco, Thermoscientific, Germany). Culture flasks were incubated at 37 °C in an atmosphere of 5% CO_2_ for 24 h to attach. On the next day, flask II was irradiated with 0.25 Gy, cells were trypsinized by 0.25%, harvested, and washed twice with PBS, and the cell pellet was suspended in DMEM media after the cell count was adjusted to 106/mL to be used for further analysis [19].

#### Irradiation process

Irradiation was performed at the National Centre for Radiation Research and Technology (NCRRT), Nasr City, Cairo, Egypt. The source of radiation was a Gamma Cell-40 (Cesium-137, ^137^Cs) with a dose rate of 1 Gy = 0.33 min, which ensures a homogeneous distribution of irradiation. Tissue-cultured cells were exposed to 0.25 Gy [13].

#### Experimental design

Human gingival fibroblasts were divided into 2 main groups: Group (I): The non-irradiated HGFs and Group (II): The HGFs irradiated with 0.25 Gy gamma radiation. Group (I) was divided into 3 subgroups: 1—the negative control group (**NC**), 2—the group in which HGFs were co-cultured with CO (**CO**), and 3—the group in which HGFs were co-cultured with CO@SNPs (**CO@SNPs**). Group (II) was divided into 3 subgroups: 1—the positive control group (**IR**), 2—the group in which HGFs were co-cultured with CO (**CO + IR**), and 3—the group in which HGFs were co-cultured with CO@SNPs (**CO@SNPs + IR**). Cell viability and microscopic examination were performed for the two groups, followed by assessment of the biological impacts.

#### Calculation of EC50 of CO and CO@SNPs on HGFs for 72 h

Non-irradiated HGFs were cultured in 96-well culture plates. 1 × 104 cells were seeded in Dulbecco’s Modified Eagle Medium (DMEM) (*Gibco, Thermoscientific, USA*), supplemented with 10% fetal bovine serum (FBS) (*Gibco, Thermoscientific, USA),* and 1% of penicillin G sodium (10,000 UI), streptomycin (10 mg), and amphotericin B (25 μg) (PSA) (*Gibco, Thermoscientific, USA*). Cells were incubated at 37 °C in an atmosphere of 5% CO_2_ for 24 h for attachment. On the next day, the media was changed, and the adherent cells were treated with serial concentrations of CO and CO@SNPs, including “0, 0.01, 0.1, 1.0, 10, and 100 µmol/mL”. The treated cells were cultured to be incubated in an atmosphere of 5% CO_2_ for 72 h. The cell growth and morphology were monitored by an inverted microscope every 24 h. The silica nanoparticles represent the clove oil carrier. At the end of incubation, the cell proliferation assay was conducted, and the percentage of viability was determined, which represents the proliferative effect of CO and CO@SNPs at the tested dilutions. The XY curve was plotted to illustrate the relation between the log doses of the agonist versus the normalized response. The best-fit point was determined by linear regression analysis for calculation of half maximal effective concentration (EC_50_). The EC_50_ was calculated using *GraphPad Prism software 9*.

#### Assessment of cell viability by cell proliferation assay (MTT)

The cell proliferation assay was performed using the *Vybrant® MTT Cell Proliferation Assay* Kit, cat. no. M6494 (ThermoFisher, Germany). When the incubation time was reached, 100 µL of media was removed and replaced by new media. Twenty microliters of 4,5-dimethylthiazol-2-yl)-2,5-diphenyltetrazolium bromide (MTT) solution (1 mg/mL) (Invitrogen, Thermoscientific, Germany) was added to each well, and the plates were incubated at 37 °C and 5% CO_2_ for 4 h. Finally, the MTT solution was removed, and 100 μL of sodium dodecyl sulfate with hydrochloric acid (SDS-HCL) was added to the wells. Cell viability was determined by measuring the optical density at 570 nm on a spectrophotometer (ELx 800; Bio-Tek Instruments Inc., Winooski, VT, USA).

#### Cell count by trypan blue stain using hemocytometer of HGFs after treatment with EC50 of CO and CO@SNPs for 72 h

One day before the experiment, 1 × 104 HGFs were seeded in a 96-well plate for 24 h to attach. Two forms of cells were assigned: non-irradiated cells and cells exposed to 0.25 Gy of ionizing radiation. Cells were maintained in Gibco Dulbecco’s Modified Eagle Medium (DMEM) (Gibco, Thermoscientific, Germany) containing 10% fetal bovine serum (FBS) (Gibco, Thermoscientific, Germany) and 1% of penicillin G sodium (10,000 IU), streptomycin (10 mg), and amphotericin B (25 μg) (PSA) (Gibco, Thermoscientific, Germany). The culture plate was incubated at 37 °C in an atmosphere of 5% CO_2_. On the next day, cells were treated individually with 1.88 µmol of clove oil and 0.172 µmol of CO@SNPs and incubated for 72 h at 37 °C in an atmosphere of 5% CO_2_. The percentage of cell death was determined by counting the cells after being stained with trypan blue. The HGFs were counted by hemocytometer to estimate the total cell count and viability percentage.

#### Gene expression analysis by quantitative real-time PCR for HGFs after treatment with EC50 of CO and CO@SNPs for 72 h

##### Purification of total RNA from harvested cells using the RNeasy® Mini Kit

Up to 1 × 106 cells were disrupted and homogenized by bead-milling in a guanidine-thiocyanate-containing lysis buffer. After addition of ethanol, the sample was loaded onto RNeasy Mini spin column. Total RNA binds to the RNeasy silica membrane, contaminants were efficiently washed away, and high-quality RNA was eluted in RNase-free water. The RNA extraction and purification was performed using RNeasy Mini kit, cat. no.: 74004, Qiagen, Hilden, Germany. The process was conducted according to the manufacturer’s protocol.

##### Reverse transcription

The reverse-transcription step was performed by the QuantiTect Reverse Transcription Kit, cat. No.: 205310 (Qiagen, Hilden, Germany). The reverse-transcription master mix was prepared on ice in a total volume of 20 µL, which was composed of 1 µL of QuantiTect Reverse Transcriptase enzyme, 4 µL of RT buffer, 1 µL of RT primer mix, and 14 µL of genomic DNA. The reaction mix was mixed and then kept on ice. The reverse-transcription master mix contains all components required for first-strand cDNA synthesis except template RNA. The reaction mix was incubated for 15 min at 42 °C, and then, it was incubated for 3 min at 95 °C to inactivate QuantiTect Reverse Transcriptase. The reverse-transcription reactions were placed on ice, and then, real-time PCR proceeded directly.

##### Gene expression analysis using SYBR-green-based PCR

Three genes were amplified from cDNA using the QuantiTect Syber Green PCR kit, cat. no.: 204141 (Qiagen, Hilden, Germany), and oligo-specific primer sequences. The genes were amplified from cDNA using the QuantiTect primer assay primer assays, cat. no.: 249900; [Hs_C-Myc, ID: QT00035406, Hs_NF-kβ, ID: QT00396823 and Hs_MAPK, ID: QT02589314]. Primer assays: The genes were amplified by QuantiTect Syber Green Master Mix (Qiagen, Hilden, Germany). The β-actin (Hs_ACTB), ID: QT000954231 primer assay was used as a housekeeper gene. All samples were analyzed using the 5-plex Rotor-Gene PCR Analyzer (Qiagen, Germany). The 2-ΔΔCT method was conducted for the analysis of gene expression levels, using β-actin as an endogenous reference control for normalization purposes.

#### Measurement of antioxidant capacity for HGFs after treatment with EC50 of CO and CO@SNPs for 72 h

Following the treatment of HGFs with the calculated EC50 of CO and CO@SNPs for 72 h, the level of lipid peroxidation, malondialdehyde (MDA), was measured using the endpoint calorimetric assay kit [malonaldehyde (MDA) colorimetric assay kit, catalog No. E-BC-K025-S, Elabscience Biotechnology, A]. In addition, the enzymatic activity of catalase (CAT) was measured using calorimetric commercial kits [Catalase (CAT) Activity colorimetric kit, cat. no. E-BC-K031-S, Elabscience Biotechnology, USA].

#### In vitro wound-healing assay “cell migration assay”

To evaluate the proliferative effect of CO and CO@SNPs at the EC50 concentration on non-irradiated HGFs in group (I) compared to irradiated HGFs in group (II), an in vitro wound-healing assay “scratch assay” was conducted. As a primary step, the cells were cultured to form a confluent cell monolayer. At this step, the monolayer represents the in vivo conditions of “intact epithelial cells” of the tissue before wounding. After the cells had become confluent, a cell-free gap in the monolayer was performed by mechanical scratching (scratch wound). The cells were treated with the EC50 calculated before at 72 h. In addition, the carrier solvent (0.1% DMSO) was used for control cells. The treated cells were incubated at 37 °C in an atmosphere of 5% CO_2_ for 72 h, and then, the healing effect was evaluated by microscopic examination. The images of HGFs were captured by a Labomed inverted microscope (Labomed, USA) and Vega Digital Camera. The magnification power was 10×, and the scale bar was 20 µmol/mL [20]. At the end of the incubation time, the relative wound density “the ratio of the occupied area in the gap to the total area in the initial gap”, the wound area (µm) “cell-free area”, and the gap width (µm^2^) “average distance between the edges of the gap” were measured over time, and the data were expressed as a percentage. The ratio of the occupied area in the gap to the total area in the tested group was normalized to the initial gap. The analysis was performed using the live-cell imaging software (Labomed, Los Angeles, USA) and LC-6 USB 3.0 Colorful CMOS Digital Cameras, Labomed, Los Angeles, USA.

#### Hematoxylin and eosin (H&E) staining of HGFs

Following the removal of the culture media, cells underwent two PBS washes. The samples were fixed with a 3.7% formaldehyde solution for 15 min at room temperature (RT). Following three rounds of PBS washing, cells were permeabilized using a 0.2% Triton X-100 solution. The cells were subsequently cleaned twice with distilled water and three times with PBS. At RT, hematoxylin was treated for 1 min. After 45 s of eosin treatment, cells were rinsed with tap water to get rid of hematoxylin. To eliminate the eosin dye, immersion in 95% and 100% ethanol was employed. Finally, the non-irradiated and irradiated HGFs were co-cultured with 1.88 µmol/mL of CO or 0.172 µmol/mL of CO@SNPs for 72 h for microscopic examination. The microscopic examination of HGFs was performed by LABOMED inverted microscope LX400, cat. no. 9126000; US, and LABOMED camera software, USA. The magnification power was 10x, and the scale bar was 50 µmol/mL.

#### Assessment of TGF-β protein expression in HGFs by immunofluorescence staining

The HGFs were treated with EC50 of CO and CO@SNPs separately and examined for the expression of Fibroblast Growth Factor β subunit (TGF-β). Cells were fixed with warm 4% formaldehyde. Then, the cells were immune-stained with Rabbit anti-TGF-β Antibody primary antibody (Invitrogen; ThermoFisher Scientific, Hilden, Germany) and incubated overnight at 4 °C. The cells were washed with PBS and incubated with Goat Anti-Rabbit IgG H&L secondary antibody-Alexa Flour 488 (Invitrogen; ThermoFisher Scientific, Hilden, Germany). The slide was covered with Prolong Gold Antifade Reagent (Abcam, Cambridge, UK) and mounted overnight at room temperature. The specimens were immediately examined or stored at 4 °C protected from light for long-term storage. The microscopic examination was performed using LABOMED Fluorescence microscope LX400, cat. no. 9126000; USA (Labmed; USA). The IF staining intensity was scored according to a four-tier system: 0, no staining; 1+, weak; 2+, moderate; and 3+, strong. In brief, the *H*-score of each sample was calculated as the sum of each intensity (0–3) multiplied by the percentage of positive cells (0–100%). The score ranged from 0 to 300. The median value of the *H*-score was calculated.

## Results

### Characterization of SNPs and CO@SNPs

**Fig. 1:**
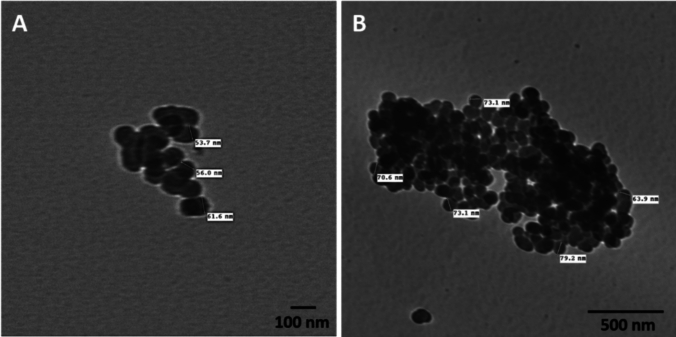
TEM images of SNPs (**A**) and CO@SNPs (**B**)

**Fig. 2:**
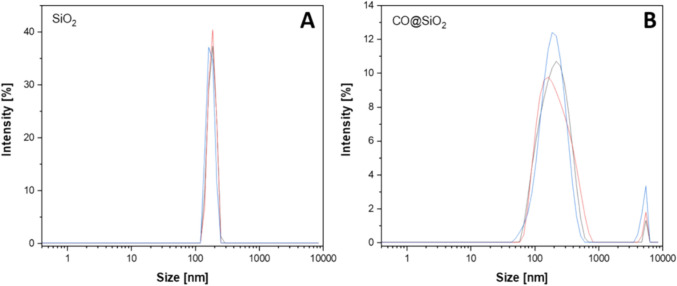
Hydrodynamic diameter of SNPs (**A**) and CO@SNPs (**B**)

The size and shape of the synthesized SNPs as well as CO@SNPs were recorded with TEM. As could be seen from (Fig. 1A, B), both SNPs and CO@SNPs were predominantly of spherical shape with an average diameter of ca. 60 nm and 70 nm, respectively. The hydrodynamic diameter (*D*_h_) of the NPs was analyzed using DLS, and the results showed that SNPs have a *D*_h_ value of 175.7 ± 23.3 nm, whereas CO@SNPs have a *D*_h_ value of ca. 208.5 ± 87.5 nm (Fig. 2A, B). The unexpected increased hydrodynamic diameter of the NPs could be ascribed to the minimal aggregation of the NPs, as could be revealed from the TEM images (Fig. 1).

**Fig. 3:**
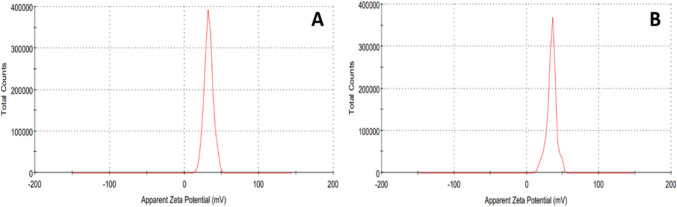
Zeta potential of SNPs (**A**) and CO@SNPs (**B**)

The obtained results showed no distinct changes in the zeta-potential values of both NPs. The Zeta potential of SNPs was found to be approximately 34.2 ± 6.09, whereas CO@SNPs have zeta-potential value of 35.0 ± 9.12 (Fig. 3).

**Fig. 4:**
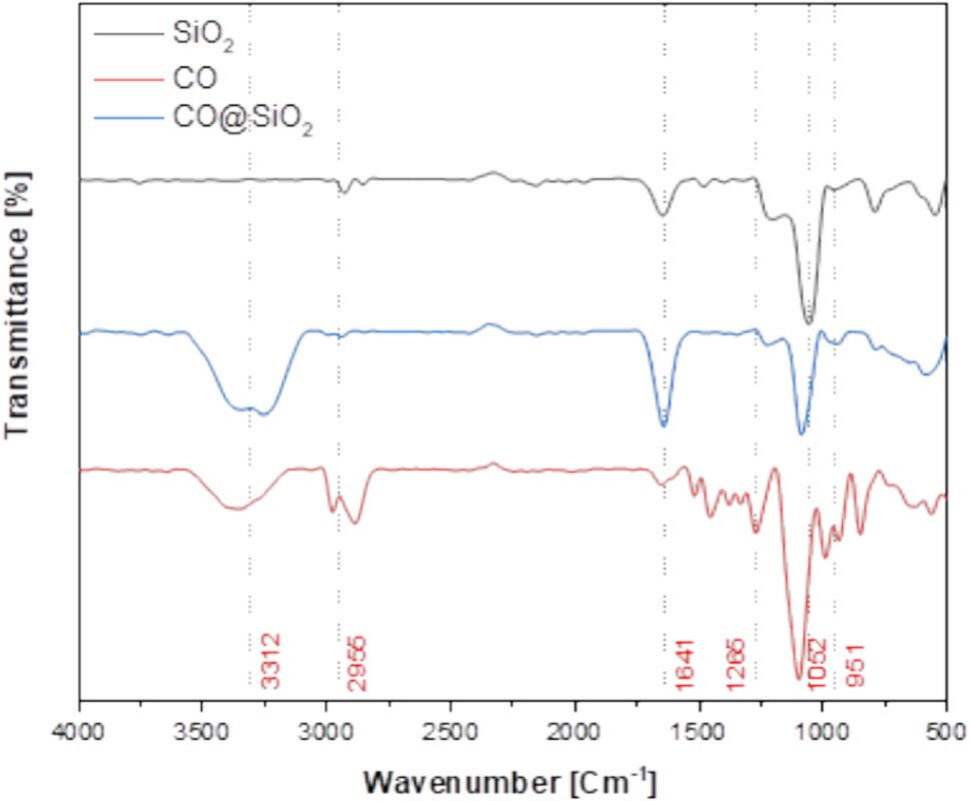
FTIR spectra of SNPs, CO, and CO@SNPs

The results of FTIR spectroscopy of original CO, SNPs, and CO@SNPs are shown in Fig. 4. FTIR analysis of SNPs showed a predominant absorption band at 1052 cm^−1^, which corresponds to the asymmetric vibration of the siloxane bond (Si–O–Si). The shoulder peak centered around ~ 950 cm^−1^ could be ascribed to the bending vibration of the Si–OH silanol group (Fig. 4). Figure 4 also shows the FTIR spectra of clove oil. The spectra show the specific absorption peaks of eugenol, the main component of clove oil, as follows: (i) between 2750 and 3500 cm^−1^,which could be ascribed to the stretching vibration of the hydroxyl (O–H) group; (ii) at ~ 1641 cm^−1^, which was due to the stretching vibration of C–H bonds in the benzene ring; (iii) at ~ 1265 cm^−1^, which could be ascribed to the stretching vibration of the C–O bonds of the phenolic hydroxyls; and (iv) at ~ 1100 cm^−1^,which corresponds to the stretching vibration of C–O–C bonds of aromatic ether. FTIR analysis of the CO@SNPs showed the specific eugenol peaks at 3312 cm^−1^, 2955 cm^−1^, 1641 cm^−1^, and 1052 cm^−1^. Significantly, the prominent peaks of SNPs, as could be seen in the spectra, mostly remained unchanged in their positions after the CO was loaded (Fig. 4). Moreover, the lack of any additional FTIR absorption peaks further confirms the successful synthesis of CO@SNPs without any alterations in the structural integrity of both CO and SNPs.

### Cytotoxicity of CO and CO@SNPs on HGFs

**Fig. 5:**
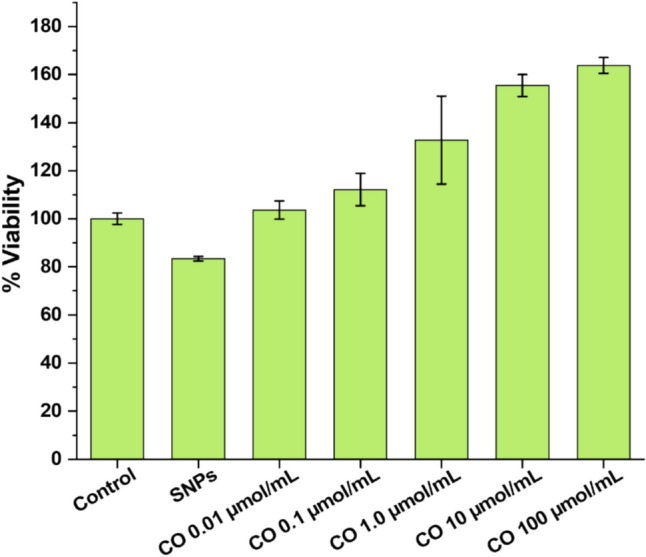
MTT assay results for Human Gingival Fibroblasts (HGFs) after treatment with serial concentrations of original CO for 72 h. *SNPs* silica nanoparticles, *CO* clove oil

**Fig. 6:**
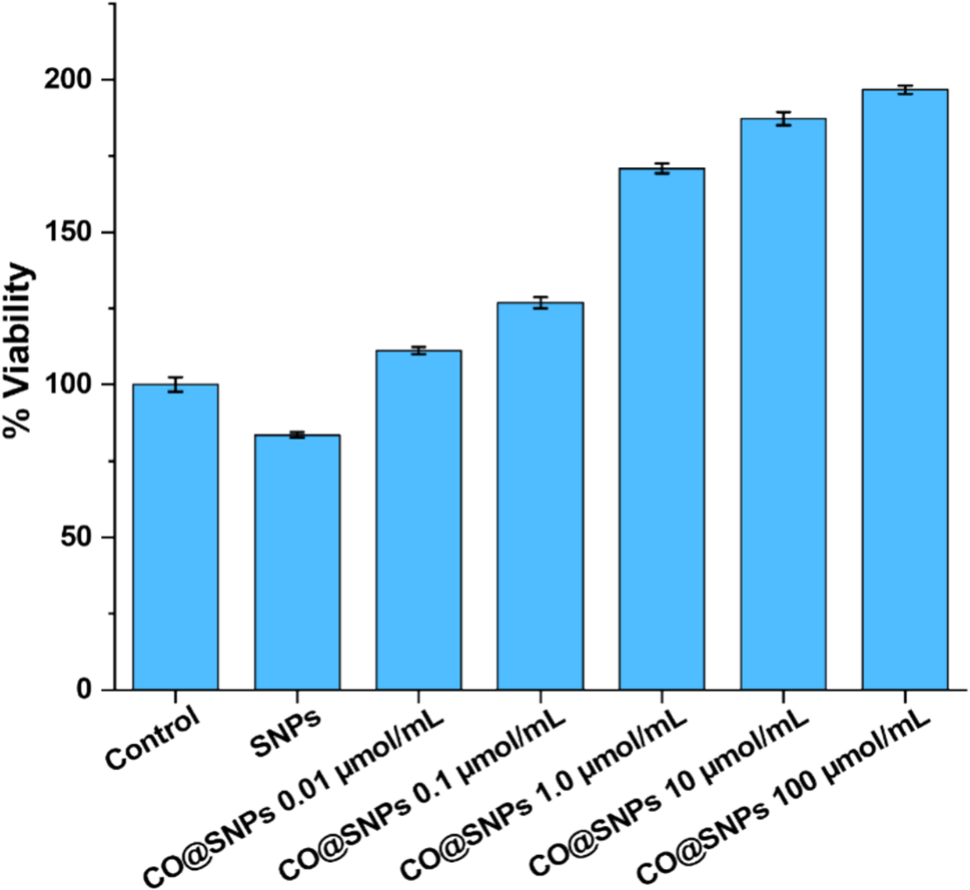
MTT assay results for HGFs after treatment with serial concentrations of CO@SNPs for 72 h. *SNPs* silica nanoparticles, *CO@SNPs* clove oil-loaded silica nanoparticles

**Fig. 7:**
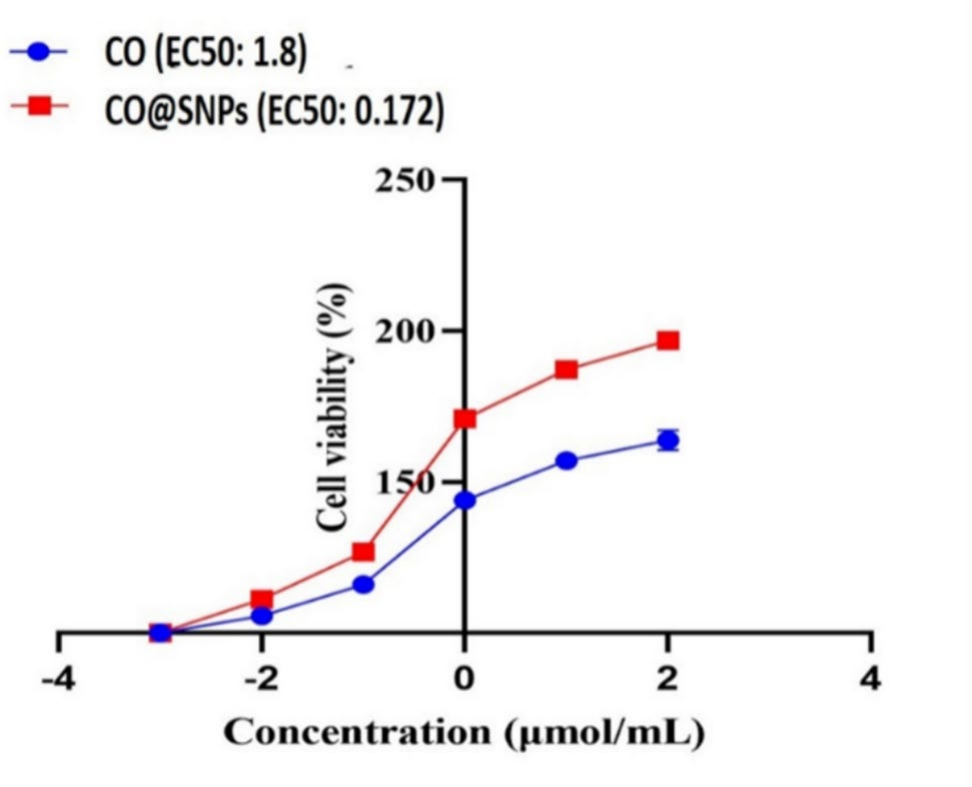
Linear regression curve illustrating the Log of EC50 for CO and CO@SNPs the normalized response in Tongue epithelial cells after treatment for 24, 48 and 72 h. *EC50* half-maximum effective concentration

Non-radiated HGFs were cultured in 96-well culture plates and treated with serial concentrations of CO and CO@SNPs, including 0, 0.01, 0.1, 1.0, 10 and 100 µmol/mL. The results of EC50 in each original compound of CO and CO@SNPs were evaluated through the average of viability percentage in different concentrations, as shown in Figs. 5, 6, and the finalized dose of EC50 was mentioned in Table 1 and Fig. 7 that illustrate the EC50 for each one in logarithmic scale for concentration versus viability percentage, where the EC50 for original CO recorded 1.89 µmol/mL and the CO@SNPs recorded 0.172 µmol/mL, that results confirm the high efficiency of CO@SNPs more than original CO, from the current results the nearly 9.1% of nanoformulation of CO give the same results of original CO.

**Table 1:** Calculation of EC50 of CO and CO@SNPs on HGFs

Log(agonist) vs. response (three parameters)		
Best-fit values	Clove oil	CO/SiO_2_ NPs
LogEC50	0.277	− 0.762
EC50	**1.88 µmol/mL**	**0.172 µmol/mL**
Bottom	76.47	− 370.3
Top	159.7	188
*R* squared	0.96	0.97

*EC50* half-maximum effective concentration

**Fig. 8:**
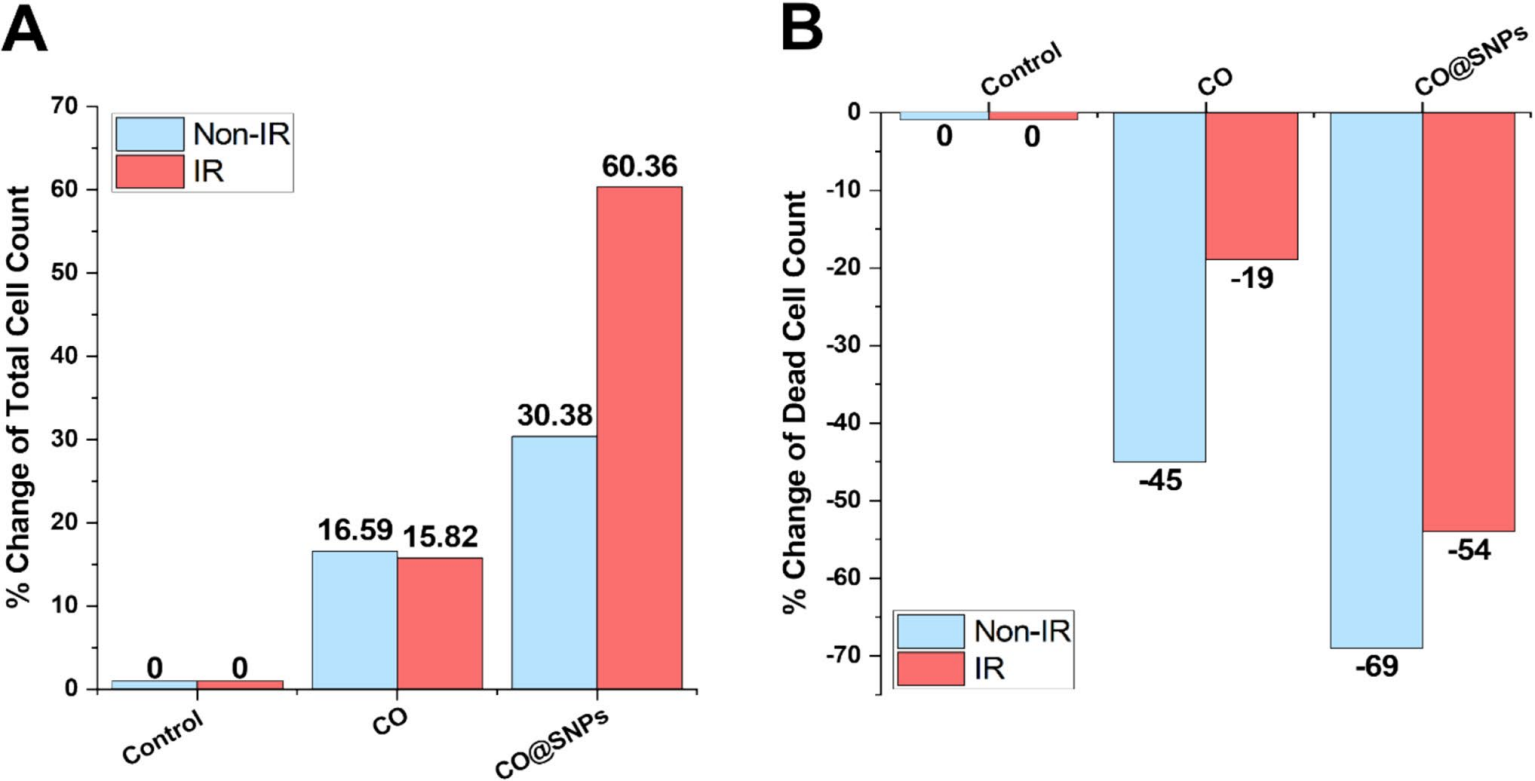
Percent change in total cell count (**A**) and dead cell count (**B**) of HGFs under the effect of CO and CO@SNPs on non-irradiated HGF cells (Non-IR) and HGF cells irradiated with low dose of gamma radiation (IR)

Human Gingival Fibroblasts growth differs according to the effect of CO in original or pure format than the effect of CO@SNPs in addition to the change in low doses of gamma irradiation (0.25 Gy of ^137^Cs). The total cell count and cell death under the effect of both types of clove oils, in addition to its effect of gamma irradiation, are shown in Fig. 8. The results approved that the irradiated cells with a low dose of gamma irradiation with its combination with CO@SNPs have nearly four times the cell growth more than pure CO singly with the same dose of gamma irradiation of HGFs, as shown in (Fig. 8), but in the case of non-radiated cells, the CO@SNPs have only 2 times more than CO singly.

### Hematoxylin and eosin (H&E) staining of HGFs

**Fig. 9:**
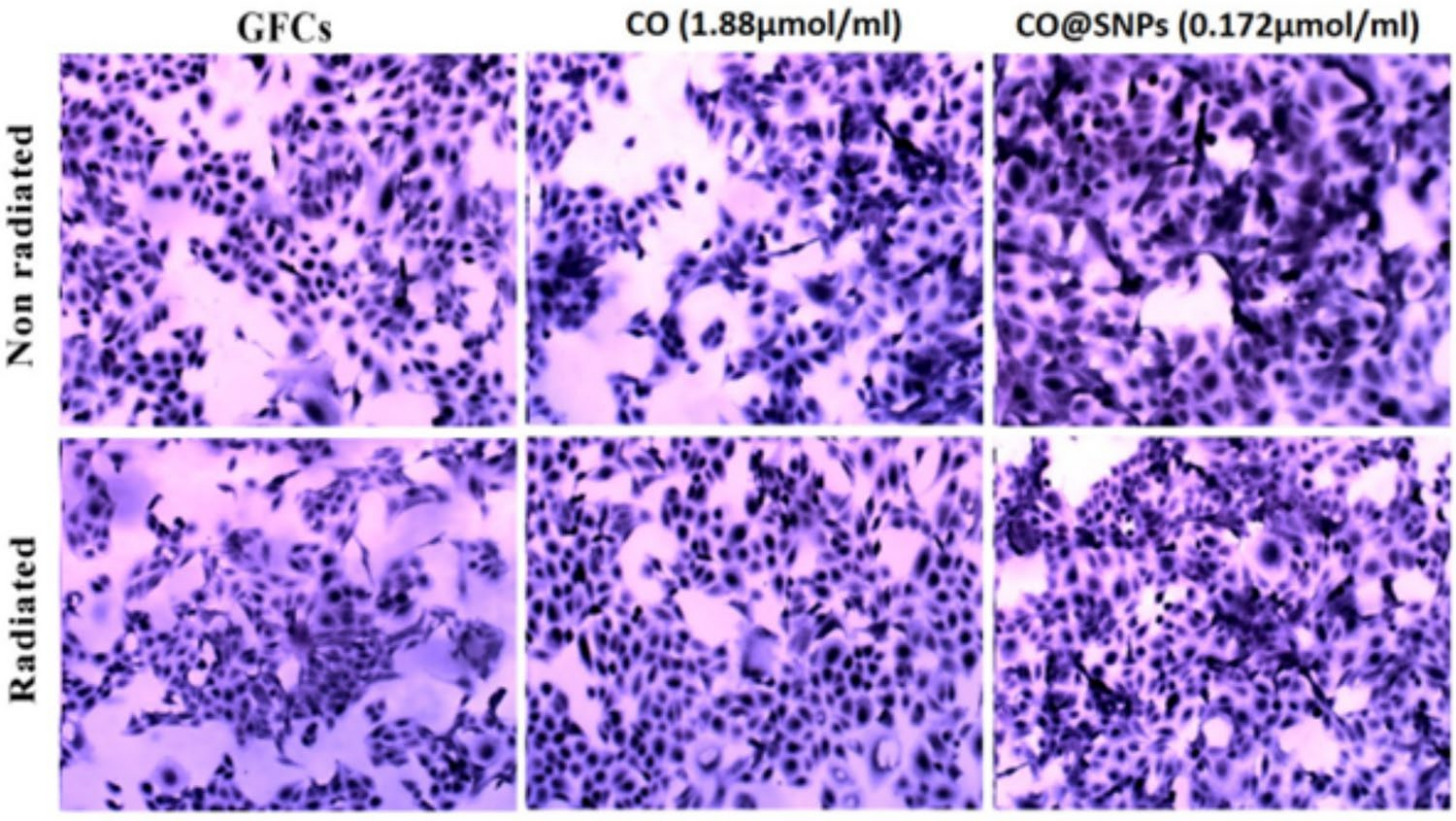
Photomicrographs illustrated the morphological changes of non-irradiated HGFs in group (I) in comparison to irradiated HGFs in group (II). Group (I) showed regular negative control HGFs with typical cell morphology and definite intercellular spaces, nearly comparable changes in HGFs co-cultured with CO, proliferative fibroblasts with less intercellular spaces and swollen hyperchromatic nuclei in HGFs co-cultured with CO@SNPs. Group (II) showed normal positive control HGFs, swollen HGFs co-cultured with CO or CO@SNPs with hyperchromatic nuclei, barely detected cell outline and hardly recognized intercellular spaces (orig. mag. ×10)

The microscopic examination of H&E-stained sections of HGFs showed that the negative control HGFs in group (I) appeared regular and adherent with normal cellular and nuclear appearance. The cells exhibited an intact cell outline with definite intercellular spaces. The HGFs co-cultured with CO appeared nearly comparable to the control group, while the HGFs co-cultured with CO@SNPs showed swollen fibroblasts associated with hyperchromatic nuclei. Less intercellular spaces were the most pronounced, indicating fibroblast proliferation. Interestingly, the morphological changes of HGFs in group (II) were more evidenced after irradiation with 0.25 Gy. Some fibroblasts showed normal appearance as in the positive control HGFs, while others were swollen with hyperchromatic nuclei and barely detected cell outline as in HGFs co-cultured with CO or CO@SNPs. Hardly recognized intercellular spaces were also noticed (Fig. 9).

### Gene expression analysis using SYBR-green-based PCR

**Fig. 10:**
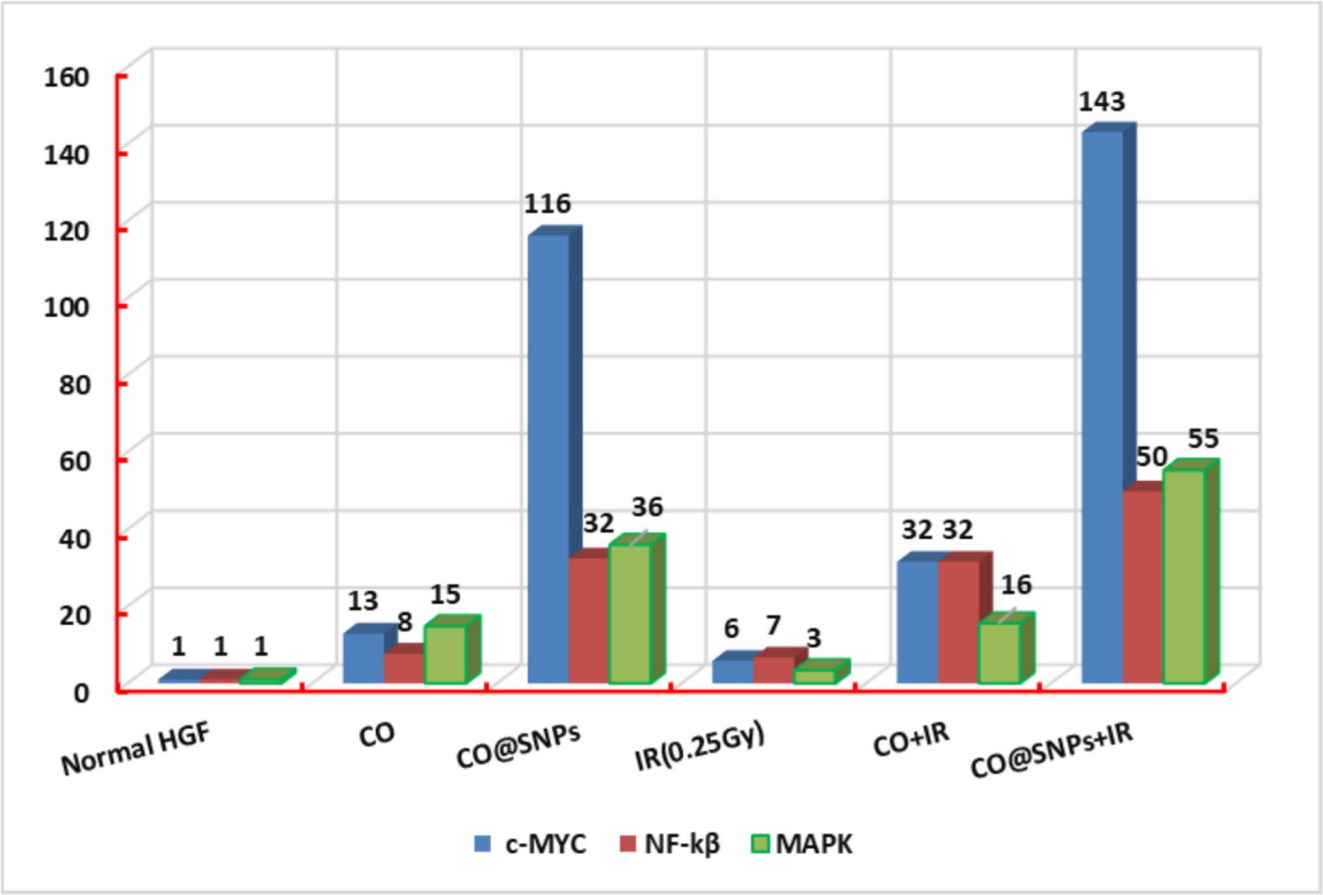
The fold of change regarding gene expression for a transcription factor that promotes cell proliferation, growth, and metabolism (c-Myc), a protein complex that controls transcription of DNA (NF-kβ), and the signaling pathway mitogen-activated protein kinase (MAPK) under the effect of CO and CO@SNPs under the normal condition and low dose of irradiation

At the level of molecular change through gene expression for some proliferative index, c-Myc, NF-kβ, and MAPK gene expressions were analyzed by real-time PCR to evaluate their changes under the effect of CO and CO@SNPs with and without a low dose of gamma irradiation. The c-Myc gene has a main role as a master regulator of cellular metabolism and proliferation, while NF-kβ is considered a transcriptional factor that is able to modulate the expression of various genes involved in a broad spectrum of cellular functions, including proliferation, survival, and migration. The Mitogen-activated protein kinases (MAPKs) regulate diverse cellular programs by relaying extracellular signals to intracellular responses to regulate cell proliferation, differentiation, motility, and survival (Table 2, Fig. 10).

**Table 2:** Gene expression for c-Myc, NF-kβ, and MAPK and their fold of change

Group	Housekeeping CT B-actin	c-MYC				NF-kβ				MAPK			
		*C*_T_	Δ *C*_T_	ΔΔ *C*_T-_	FC	C_T_	Δ *C*_T_	ΔΔ *C*_T-_	FC	*C*_T_	Δ *C*_T_	ΔΔ *C*_T-_	FC
HGFs	26.84	31.6	4.71	0	1	30.3	3.47	0	1	30.5	3.68	0	1
CO	31.27	32.3	1.02	− 3.69	12.91	31.8	0.52	− 2.95	7.73	31.1	− 0.2	− 3.89	14.8
CO@SNPs	32.74	30.6	− 2.2	− 6.86	116.2	31.2	− 1.6	− 5.02	32.5	31.3	− 1.5	− 5.17	36
Irradiated (0.25 Gy)	28.05	30.2	2.16	− 2.55	5.86	28.8	0.72	− 2.75	6.73	30	1.9	− 1.78	3.43
CO + IR	31.55	31.3	− 0.3	− 4.98	31.56	30	− 1.5	− 4.98	31.6	31.3	− 0.3	− 3.96	15.6
CO@SNPs + IR	32.29	29.8	− 2.5	− 7.16	143	30.1	− 2.2	− 5.64	49.9	30.2	− 2.1	− 5.79	55.3

*Ct* cycle threshold, *Δ* delta, *FC* fold change of each gene after normalization to untreated HGFs with β-actin housekeeper gene

### Measurement of antioxidant capacity in HGFs after treatment with EC50 of CO and CO@SNPs for 72 h

This study evaluates the impact of various treatments (CO, CO@SNPs, IR, CO + IR, and CO@SNPs + IR) on two oxidative stress markers: malondialdehyde (MDA) and catalase activity in human gingival fibroblasts (HGFs).

**Malondialdehyde (MDA)**

**Fig. 11:**
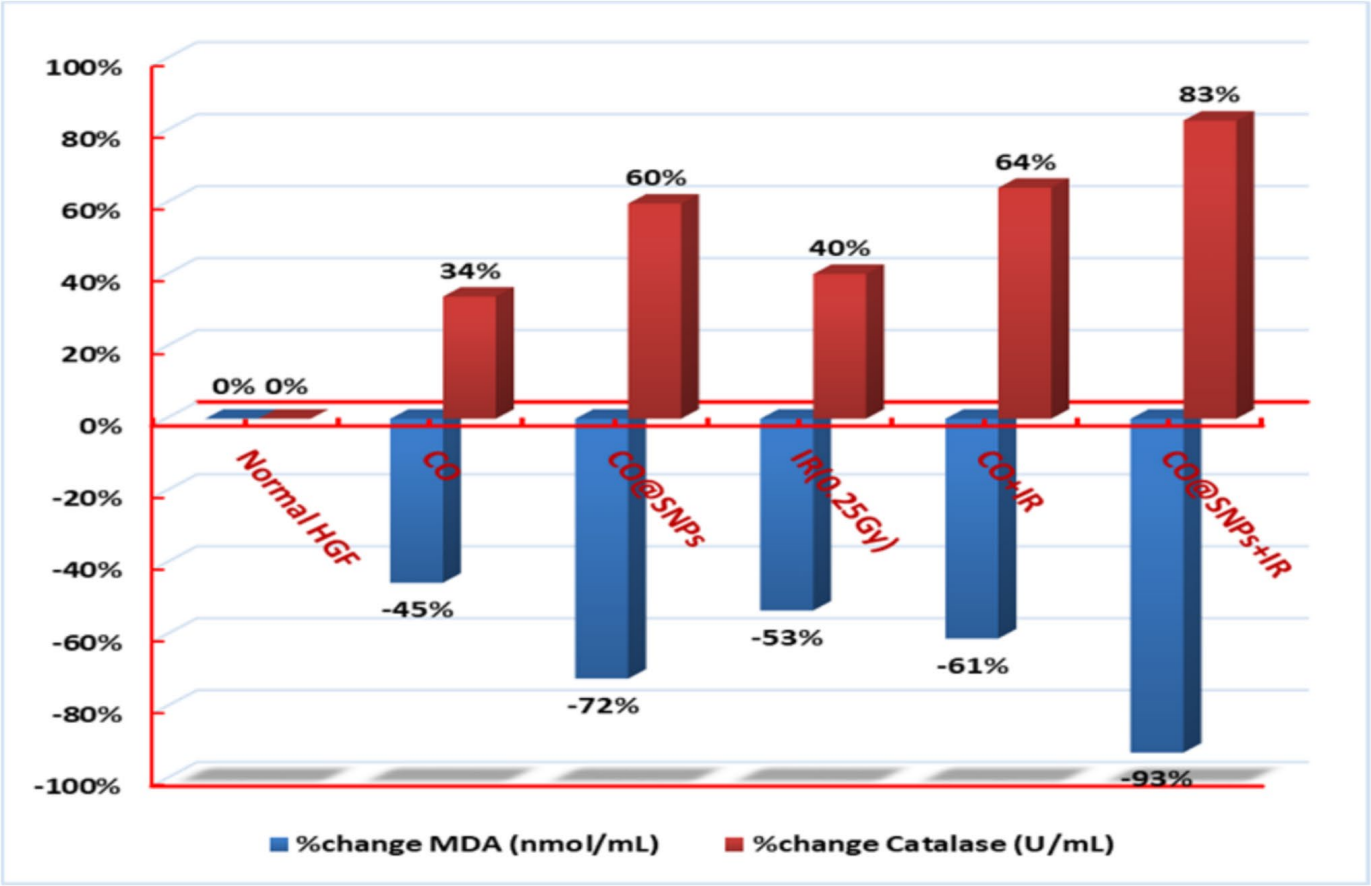
Percentage change in MDA levels and catalase activity in different groups

MDA, a marker of lipid peroxidation, is widely utilized as an indicator of oxidative stress. In the control group (NC HGFs), the MDA level was measured at 9.94 nmol/mL, serving as the baseline (0% change). Treatment with CO resulted in an MDA level of 5.42 nmol/mL, corresponding to a 45% reduction, while the CO@SNPs group exhibited a further reduction to 2.77 nmol/mL (72% decrease), indicating that CO@SNPs is more effective in mitigating oxidative stress compared to CO alone. Exposure to low-dose radiation (IR group) also reduced MDA levels to 4.65 nmol/mL (53% decrease), demonstrating its capacity to lower oxidative stress. Notably, in the CO + IR and CO@SNPs + IR groups, MDA levels were reduced to 3.88 nmol/mL (61% decrease) and 0.72 nmol/mL (93% decrease), respectively, highlighting a synergistic effect of the combined treatments in reducing oxidative stress (Table 3, Fig. 11).

**Table 3:** MDA levels and catalase activity in the tested groups

Groups	MDA (nmol/mL)		Catalase (U/mL)	
	Mean ± SE	%Change	Mean ± SE	%Change
NC HGFs	9.94 ± 0.71	0%	317.1 ± 16.4	0%
CO	5.42 ± 0.28^a^	− 45%	424.81 ± 26.97^a^	34%
CO@SNPs	2.77 ± 0.14^ab^	− 72%	506.76 ± 36.21^ab^	60%
IR	4.65 ± 0.31^a^	− 53%	444.75 ± 29.46^a^	40%
CO + IR	3.88 ± 0.18^abc^	− 61%	520.75 ± 32.96^ab^	64%
CO@SNPs + IR	0.72 ± 0.03^abc*d*^	− 93%	579.84 ± 38.67^abc^	83%

^a^High significant versus to normal control human gingival cells (NC HGFs), *p* value < 0.01

^b^High Significant versus to Clove Oil group (CO), *p* value < 0.01

^C^Significant vs to CO@SNPs, C*: high significant vs to CO@SNPs

^d^Significant to CO + IR group, d*: high significant vs to CO + IR

**Catalase activity**

Catalase, an enzyme responsible for mitigating oxidative stress by catalyzing the breakdown of hydrogen peroxide into water and oxygen, showed significant variations in activity across treatment groups. In the control group (NC HGFs), catalase activity was measured at 317.1 U/mL (baseline). In the CO + IR and CO@SNPs + IR groups, catalase activity increased to 520.75 U/mL (64% increase) and 579.84 U/mL (83% increase), respectively, indicating a pronounced enhancement in antioxidant defense. The CO@SNPs + IR group demonstrated the most substantial improvement, suggesting a strong synergistic effect of CO@SNPs combined with low-dose radiation on catalase activity (Table 3, Fig. 11).

### In vitro wound-healing assay

**Fig. 12:**
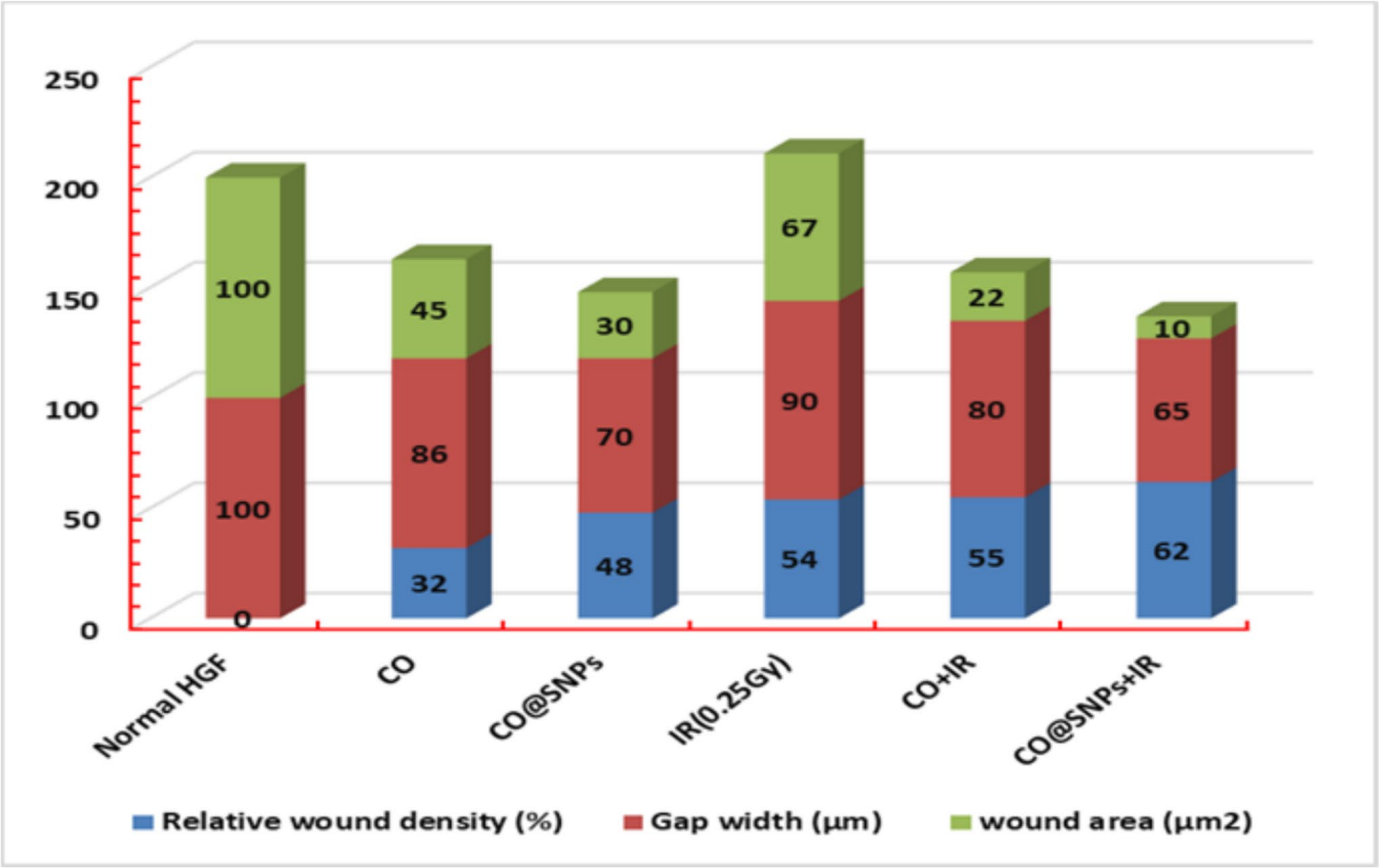
Change in relative wound density, wound area, and gap width in Co and CO@SNPs singly or combined with a low dose of radiation

**Fig. 13:**
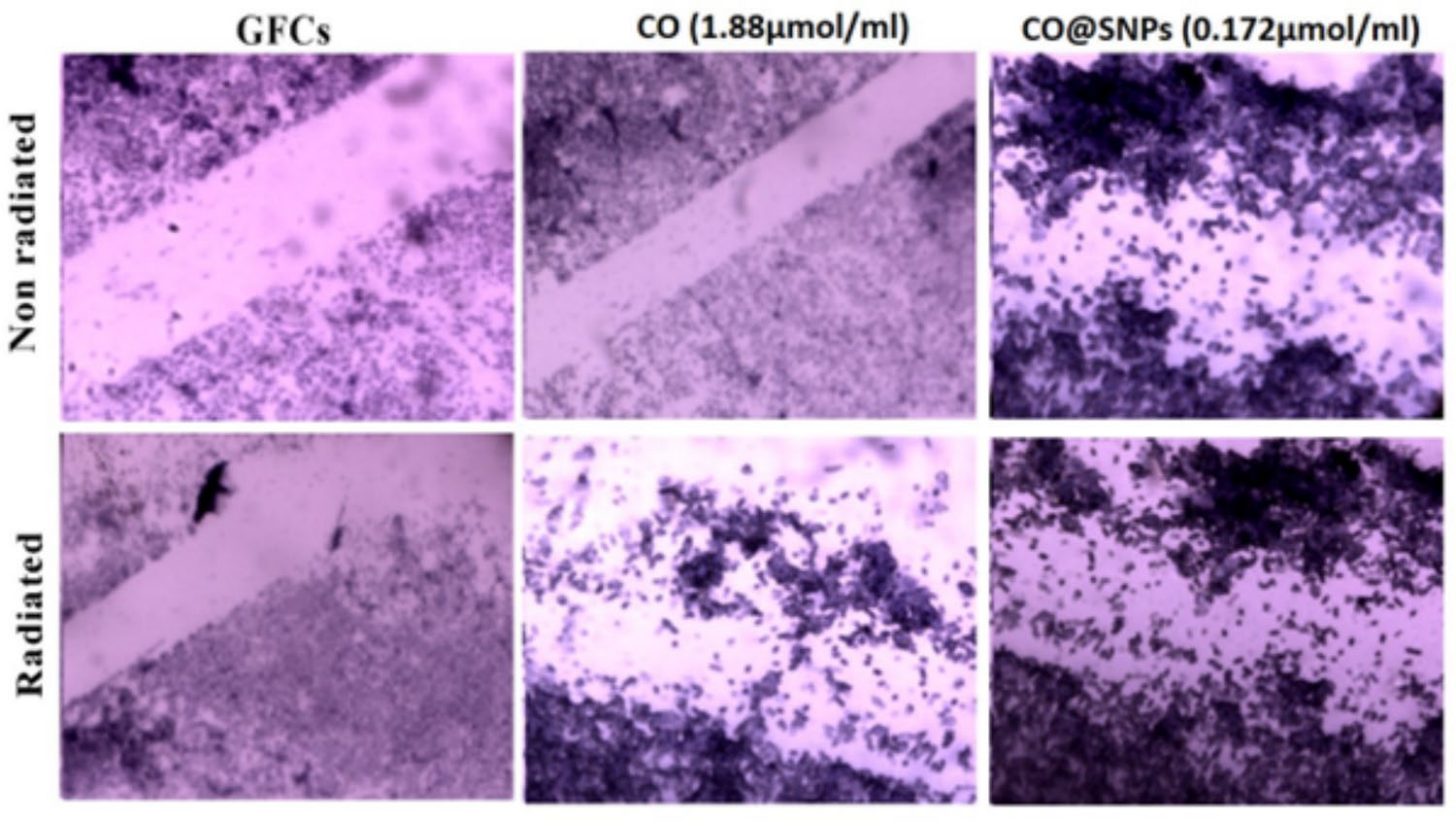
Photomicrographs illustrating the proliferative effect of CO and CO@SNPs at the EC50 concentration on the closure of wound in vitro [non-irradiated HGFs in group (I) compared to irradiated HGFs in group (II)]. Group (I) showed wide gap between the two edges of the scratch in the negative control HGFs, roughly proliferative fibroblasts, and mild closure of gap between the two edges of HGFs cultured in DMEM and supplemented with CO which was improved in HGFs supplemented with CO@SNPs. Group (II) showed comparable results in the positive control HGFs to the negative control HGFs in group (I), near complete closure of wound with severe infiltration of HGFs between the two edges of the scratch in HGFs cultured in DMEM and supplemented with CO and CO@SNPs, respectively (orig. mag. ×10)

The in vitro proliferative effect of CO and CO@SNPs at the EC50 concentration on the closure of wound was evaluated. The microscopic examination of HGFs was performed using light inverted microscope. Examination of the HGFs in group (I) showed that the negative control HGFs appeared with wide gap between the two edges of the scratch. On the other hand, the non-irradiated HGFs cultured in DMEM and supplemented with CO showed roughly proliferative fibroblasts and mild closure of the gap between the two edges, which was improved in the HGFs cultured in DMEM and supplemented with CO@SNPs for 48 h. The positive control HGFs in group (II) showed nearly the same results as the HGFs in the negative control group (I). In contrast, irradiated HGFs cultured in DMEM and supplemented with CO for 48 h showed near complete closure of the wound with severe infiltration of HGFs between the two edges of the scratch, which was accentuated in the HGFs supplemented with CO@SNPs (Figs. 12, 13).

The wound-healing assay showed significant differences in the percentage of relative wound density, gap width, and wound area between control HGFs, HGFs cultured in DMEM and supplemented with CO, and those supplemented with CO@SNPs at the EC50 concentration for 72 h in the studied groups, respectively. Moreover, the low dose of gamma irradiation (0.25 Gy) enhanced the proliferative effect of CO and CO@SNPs at the EC50 concentration on the HGFs and showed the best wound-healing effect. The gap width showed the greatest distance in either non-irradiated or irradiated control HGFs (100 µm, 90 µm), followed by those supplemented with CO (86 µm, 80 µm), and then those supplemented with CO@SNPs (70 µm, 65 µm) at the EC50 concentration in the studied groups. As expected, the wound area showed the same results too (Table 4).

**Table 4:** In vitro wound-healing assay results measured in cultured HGFs after treatment with the EC50 of CO and CO@SNPs for 72 h

Serial	Group	Relative wound density (%)	Gap width (µm)	Wound area (µm^2^)
1	HGFs	0	100	100
2	CO	32	86	45
3	CO@SNPs	48	70	30
4	IR (0.25 Gy)	5.4	90	67
5	CO + IR	55	80	22
6	CO@SNPs + IR	62	65	10

**Relative wound density**: ratio of the occupied area in the gap to the total area in initial gap, **wound area** (µm^2^): cell-free area, and **gap width** (µm): average distance between the edges of the gap

### TGF-β protein expression in HGFs by immunofluorescence

**Fig. 14:**
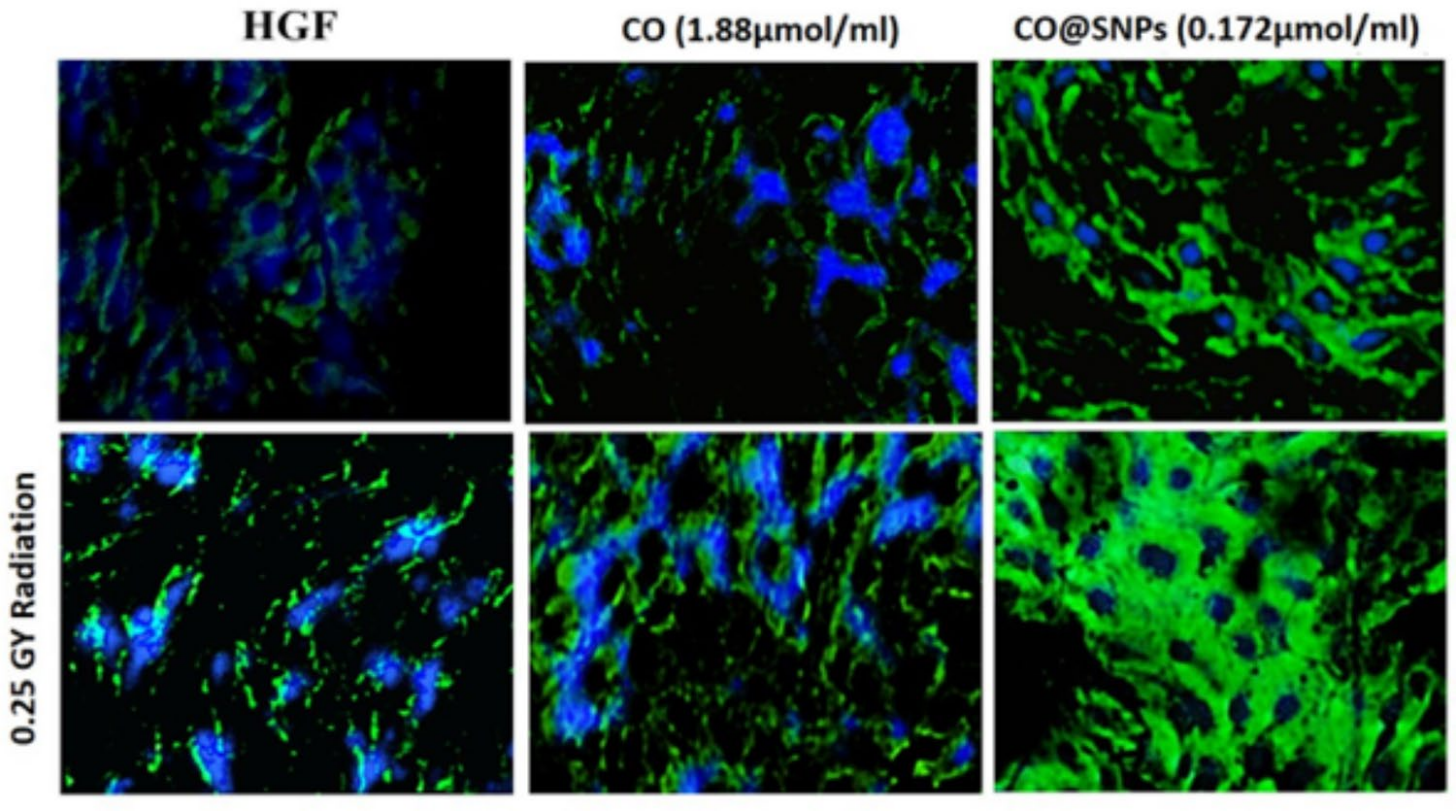
Expression of TGF-β protein in HGFs by immunofluorescence. The HGFs were co-cultured with 1.88 µmol/mL of clove oil or 0.172 µmol/mL of CO@SNPs, for 72 h to evaluate their healing effect on HGFs. The untreated HGFs represent the negative control. The Immunofluorescence stain with specific polyclonal antibodies against TGF-β (green) shows the expression of TGF-β as a promoting proliferation factor during wound healing and confirms The CO and CO@SNPs efficiency in stimulating HGFs proliferation

The IF staining intensity was scored according to a four-tier system, and the *H*-score was calculated where the harvested HGFs were examined by immunofluorescence staining with specific polyclonal antibodies against TGF-β (green). An AlexFluor 488 anti-rabbit IgG secondary antibody was used for detection. Fluorescence was examined by an immunofluorescence microscope and scored as in Table 5 and Fig. 14**.**

**Table 5:** Calculation of *H*-score for TGF-β protein expression in cultured HGFs after treatment with the EC50 of CO and CO@SNPs for 72 h with and without irradiation

No.	Code	HGFs stained with TGF-β		
		% of positive cells	Staining intensity	*H*-score
1	Normal HGF	45	1	45
2	CO	62	1	62
3	CO@SNPs	78	2	156
4	IR(0.25 Gy)	55	1	55
5	CO + IR	75	1	75
6	CO@SNPs + IR	82	2	164

*H*-score of each sample was calculated as the sum of each intensity (0–3) multiplied by the percentage of positive cells (0–100%). The score ranged from 0 to 300. The median value of *H*-score was calculated

0, no staining; 1+ , weak; 2+ , moderate; and 3+ , strong

## Discussion

Using nanotechnology in clinical dentistry will be crucial to improving oral healthcare and reducing the effort for dentists. Innovative nanotechnological approaches effectively tackle dental issues, and it is believed that using nanomaterials can yield greater success than using standard materials. Moreover, patients are drawn to dentistry through nanodentistry, because it is time and money-efficient and gives more effective results. The creation of modified nanomaterials will undoubtedly aid in resolving the most dental issues. Although the effects of nanotechnology in treating oral diseases are limited, research in this field is moving quickly forward, making advancements conceivable soon [21].

Recently, the essential oils derived from various plant sources have revolutionized dentistry and other domains of medical science. Most remarkably, it has been claimed that clove oil (CO), taken from the dried buds of *Eugenia Caryophyllata*, can reduce pain and speed up recovery. Eugenol, a primary phenolic component of CO, is thought to be responsible for several important pharmacological advantages, including pulp-sedating, obtunding, anti-inflammatory, antibacterial, and antifungal qualities [22]. Although the use of CO attained commercial success, several clinical concerns emerged regarding the hampering effect of eugenol. Moreover, pure eugenol, at elevated concentrations, exhibited concentration-dependent toxicity toward mammalian cells. Considering the toxic effect of eugenol and the highly volatile nature of CO, efforts were directed to encapsulate CO in nanosized, biocompatible, biodegradable delivery vehicles [23]. Using CO, applied with new nanotechnologies, allows the successful development of novel nanoparticles with superior properties and greater therapeutic power in clinical dentistry [24]. De Oliveira and Tavares [25] documented that nanoencapsulation of CO resulted in controlled release, increased cell viability and blood compatibility, and enhanced its characteristics compared to native oil. This difference may have a substantial impact on their biological activities and potential health benefits [25].

Compared to the in vivo test systems, in vitro biocompatibility testing has numerous advantages. When materials come into touch with or are introduced into living cells, these tests can mimic the biological reactions to those compounds. Compared to in vivo trials, the conditions of these experimentally controlled tests are more strictly standardized. Due to their speed, simplicity, and lack of ethical issues, these tests are better suited for researching novel materials than expensive and time-consuming animal investigations [26]. The present work utilized the cell culture technique of HGFs because of their cost-effectiveness, repeatability, and relevance to clinical circumstances. They also have the privilege of being easily isolated and growing quickly in standard culture medium. Cytotoxicity tests reveal that these cells are extremely susceptible. Thus, the current study prepared a suitable size of CO@SNPs and selected concentrations to in vitro evaluate the regenerative effect of clove oil (CO) in its native form and its loading into silica nanoparticles (CO@SNPs) on the HGFs system, either non-irradiated or exposed to a low dose of gamma radiation, which mimics the in vivo effect of CO on human fibroblast cells, especially gingival fibroblasts [27].

For studying CO, in vitro microscopic examination and biological assessment were thoroughly conducted. The use of CO on cultured HGFs showed high cell viability after treatment with serial concentrations of clove oil for 72 h, which indicated the safety of CO usage on HGFs. This is consistent with [28], who studied the activity of CO on a dermal fibroblast system and did not find any of the four investigated CO concentrations to be overly cytotoxic; hence, CO was used for further biological analysis. An inverted light microscope and histologic examination with H&E stain were executed to detect distinct morphological changes among the different groups throughout the study period. The microscopic evaluation of negative control HGFs in Group (I) revealed typical HGFs with normal cellular and nuclear appearance, intact cell outlines, and definite intercellular spaces. The HGFs co-cultured with CO appeared nearly comparable to the negative control HGFs, and those results came in the same line with the photomicrographs of the in vitro wound-healing assay for the same subgroups. Furthermore, the in vitro wound-healing assay results revealed that the percentage of relative wound density, gap width, and wound area was the greatest in the negative control HGFs, followed by those supplemented with CO after 72 h. According to researchers [27, 28] who studied the effect of CO on cultured human fibroblasts, the mild decrease in gap width could be attributed to the anti-inflammatory, antioxidant, and tissue remodeling activity of CO and its potential significance in wound healing. The authors credited those effects to the major active component, eugenol. In addition, Banerjee et al. [29] verified the anti-inflammatory and wound-healing effects of CO after in vivo evaluation on Wistar rats, suggesting beneficial properties for future use in the healing of different tissues. On the other hand, the photomicrographs of HGFs co-cultured with CO@SNPs showed swollen fibroblasts associated with hyperchromatic nuclei and fewer intercellular spaces. The in vitro wound-healing assay declared a significant decrease in relative wound density, gap width, and wound area associated with histological criteria denoting fibroblast proliferation, indicating the powerful effect of CO@SNPs in wound healing after 72 h. The in vitro proliferative activity and wound-healing potential of CO@SNPs in this study supported findings from previous studies; the authors confirmed its healing potential using an in vitro scratch assay depending on its anti-inflammatory and antioxidant properties [30, 31]. The microscopic evaluation of HGFs in Group (II) revealed that the low dose of gamma radiation (0.25 Gy) showed synergistic proliferative effects of CO and CO@SNPs at the EC50 concentration on the HGFs and showed the optimal histological wound-healing effect, which confirmed the hormesis effect of the low dose of gamma radiation [13]. Moreover, the in vitro wound-healing assay documented the maximum decrease in relative wound density, gap width, and wound area after 72 h. Our findings are in accordance with [32, 33], who reported that 0.05 Gy of ionizing radiation enhances cell proliferation in human lung fibroblasts. Irradiated cells demonstrated increased proliferation and protein production compared to the control samples. Marconi et al. [34] explained that the low dose of irradiation might cause an initial pause, followed by a significant increase in proliferation. An initial “pause” in cell proliferation could be a protective mechanism for the cells to minimize DNA damage caused by irradiation.

Regarding the mechanism of action of CO and CO@SNps on HGFs, the gene expression of some regulatory genes that are responsible directly or indirectly for cell proliferation and division, the c-Myc gene represents a transcription factor that promotes cell proliferation, growth, and metabolism, and its upregulation correlates with increased cell division [35]. Nuclear Factor Kappa light chain enhancer of activated B cells (NF-kB) represents a protein complex that controls the transcription of DNA, which can influence immune response and cell survival; thus, its activation leads to enhanced survival and proliferation of cells in response to stimuli [36]. Mitogen-activated protein kinase (MAPK) represents a signaling pathway for cellular processes including growth, proliferation, and differentiation [37]. In the current results, the CO increases cell proliferation through upregulation of gene expression for the previous genes (c-Myc, NF-kB, and MAPK), but the CO@SNPs in combination with a low dose of gamma radiation achieve the best results in cell growth and decreasing wound. The gap where the fold change of gene expression c-Myc, NF-kB, and MAPK was recorded at 143, 49.9 and 55.3, respectively, in comparison to 31.56, 31.6, and 15.6 in the case of natural CO combined with a low dose of radiation, which represents a highly significant difference in comparison to the single usage of clove oil. For more validation of the mechanism, the expression of the TGF-β protein was evaluated by the immunofluorescence technique and imaged as mentioned in Fig. 14. Also, the combination of clove oil loaded on silica nanoparticles with a low dose of radiation achieves the high expression of TGF-β protein, which represents a transforming growth factor beta as a multifunctional cytokine that plays a crucial role in the regulation of cell proliferation, differentiation, and various cellular processes, thus contributing to tissue repair and proliferation that plays an important role in the regeneration and proliferation of gingival cell growth that supports and helps the dentist to recover some problems in oral biology regarding gingival issues.

The balance between oxidative stress (ROS) and antioxidant activities represents the healthy status for cell growth and proliferation [38]. MDA and Catalase (CAT) exhibit a complex interplay in regulating normal cell proliferation. The dual roles of MDA as a marker of ROS and a potential signaling molecule. Catalase’s protective functions as antioxidant enzymes underscore the importance of oxidative balance [39]. The proper modulation between MDA and CAT is vital for maintaining cellular homeostasis and supporting the proliferative capacity of gingival cells. The effect of CO@SNPs combined with a low dose of gamma radiation achieves significant results in increasing the CAT activity and is the best at decreasing the MDA level compared to normal human gingival cells and treated groups with CO or CO@SNPs.

These results elucidate the biocompatibility of CO@SNPs, which has the advantage of possessing a better window of maneuverability, suggesting it as a superior alternative to CO in the dental field [40]. However, it is difficult to translate the biological response observed in the in vitro tests to a clinical situation [27]. Therefore, further animal studies and clinical trials are needed to reinforce the existing evidence regarding the biological effect of CO@SNPs on different oral tissues.

## Conclusion

The current study aimed to evaluate the efficiency of clove oil (CO) singly, clove oil loaded on silica nanoparticles (CO@SNPs) with or without a low dose of gamma radiation. The experiment started with determining the EC50% dose: 1.88 µmol/mL and 0.172 µmol/mL for both CO and CO@SNPs, respectively, which confirmed the higher efficiency of nanoformulation than normal clove oil, where the EC50 dose of natural clove oil is 10.93-fold the EC50% of CO@SNPs. The validation of the current results was done by the viability percentage and total cell count followed by Hematoxylin and Eosin (H&E) staining of HGFs that reveals the cells treated with CO@SNPs + IR with hyperchromatic nuclei, barely detected cell outlines and hardly recognized intercellular spaces. The mechanism of action is shown through the gene expression of some genes responsible for cell proliferation and metabolism evaluated through real-time PCR for c-MYC, NF-kβ, and MAPK. Their results proved the high efficiency of CO@SNPs with a low-dose gamma radiation compared to the single treatment with CO and CO@SNPs. We consider the CO@SNPs without gamma radiation to have a high accident rate in comparison to CO singly. On the method of validation of the protein level expression using immunofluorescence, the expression of TGF-β protein on HGFs was evaluated. It confirmed the previous result where the H-score recorded 164 in treated cells with CO@SNPs & a low-dose radiation that represents 3.64-fold in untreated cells and 2.65-fold in the clove oil-treated group. Due to the role of oxidative stress and antioxidant enzyme activities in cell homeostasis, the group treated with CO@SNPs & LD of IR has a change percentage of − 93% reduction in MDA level and + 83% increase in CAT activity compared to untreated cells. Finally, the wound-healing assay was performed by scratch assay. The wound gap width recorded the lowest value, 65 µm, in treatment with CO@SNPs with radiation and recorded 70 µm without radiation, but in the case of treatment with clove oil in natural form, it recorded 80 µm with radiation and 86 µm without radiation at the same conditions. At the end of this study, we recommend the topical usage of CO@SNPs in the oral cavity to recover any damage or to enhance the regenerative activity targeting the gingival cells after any minor dental interventions. After the significant results of loading clove oil in silica nanoparticles in vitro, it is also recommended to use CO@SNPs in more invasive protocols as an adjunct treatment, particularly for individuals undergoing treatments that affect the periodontium, due to their higher safety profile compared to other chemical agents commonly used. In light of these findings, it is recommended to include CO@SNPs in treatment protocols aiming at promoting and enhancing gingival health. However, further clinical trials are recommended to standardize its applications, determine the in vivo optimal dosages, and evaluate the long-term effects.

## Data Availability

All the data generated or analyzed during this study are included in this published article.
